# Activated monocytes resist elimination by retinal pigment epithelium and downregulate their OTX2 expression via TNF‐α

**DOI:** 10.1111/acel.12540

**Published:** 2016-09-22

**Authors:** Thibaud Mathis, Michael Housset, Chiara Eandi, Fanny Beguier, Sara Touhami, Sacha Reichman, Sebastien Augustin, Pauline Gondouin, José‐Alain Sahel, Laurent Kodjikian, Olivier Goureau, Xavier Guillonneau, Florian Sennlaub

**Affiliations:** ^1^ Institut de la Vision17 rue Moreau75012ParisFrance; ^2^ UPMC University of Paris 06INSERMCNRSSorbonne UniversitésParisFrance; ^3^ Department of OphthalmologyCroix‐Rousse University HospitalHospices Civils de LyonUniversity of medicine Lyon 1103 Grande rue de la Croix Rousse69317Lyon Cedex 04France; ^4^ Department of Clinical ScienceEye ClinicUniversity of TorinoTorinoItaly

**Keywords:** age‐related macular degeneration, monocytes, OTX2, retinal pigment epithelium, TNF‐α, visual cycle

## Abstract

Orthodenticle homeobox 2 (OTX2) controls essential, homeostatic retinal pigment epithelial (RPE) genes in the adult. Using cocultures of human CD14^+^ blood monocytes (Mos) and primary porcine RPE cells and a fully humanized system using human‐induced pluripotent stem cell‐derived RPE cells, we show that activated Mos markedly inhibit RPE *OTX2* expression and resist elimination in contact with the immunosuppressive RPE. Mechanistically, we demonstrate that TNF‐α, secreted from activated Mos, mediates the downregulation of OTX2 and essential RPE genes of the visual cycle among others. Our data show how subretinal, chronic inflammation and in particular TNF‐α can affect RPE function, which might contribute to the visual dysfunctions in diseases such as age‐related macular degeneration (AMD) where subretinal macrophages are observed. Our findings provide important mechanistic insights into the regulation of OTX2 under inflammatory conditions. Therapeutic restoration of OTX2 expression might help revive RPE and visual function in retinal diseases such as AMD.

## Introduction

Orthodenticle homeobox 2 (OTX2) is a key transcription factor for the development of the brain and sensory organs (Acampora *et al*., 1995; Cantos *et al*., 2000; Fossat *et al*., 2006). Mutations of *OTX2* in humans are associated with ocular malformation, pituitary defects, and mental retardation (Gorbenko Del Blanco *et al*., 2012). During retina development, OTX2 controls the specification of the retinal pigment epithelium (RPE), a monolayer of pigmented cells adjacent to the photoreceptors outer segments (POS). OTX2 expression is maintained in the adult RPE where it regulates the expression of a number of essential genes such as tyrosinase, and P protein, necessary for RPE melanogenesis to inhibit light back scatter (Martínez‐Morales *et al*., 2003; Housset *et al*., 2013), and transferrin (TRF), an essential iron transporter (Housset *et al*., 2013). It also regulates genes crucially involved in the retinol visual cycle: transthyretin (TTR), a retinol carrier, and retinol dehydrogenase 5 (RDH5) that re‐isomerizes all‐trans‐retinal into 11‐cis‐retinal (Housset *et al*., 2013). Complete OTX2 ablation in adult mice induces progressive photoreceptor degeneration (Housset *et al*., 2013). Reduced OTX2 in RPE cells and therefore TTR and RDH5 expression likely leads to the impaired capacity to import and revert all‐trans‐retinal into 11‐cis‐retinal, therefore slowing the visual cycle, which increases the recovery time after bleach, as observed in RDH5‐deficient patients (Cideciyan *et al*., 2000). Interestingly, the early stages of age‐related macular degeneration (AMD, a major cause of visual impairment) are also associated with a marked increase in recovery time after bleach before significant loss of RPE or photoreceptors occur (Owsley *et al*., 2001; Flamendorf *et al*., 2015), which suggests that the visual cycle is slowed before degeneration appears. The molecular reasons for these dysfunctions are unknown.

The RPE also expresses immunosuppressive factors, such as Fas ligand (FasL), that lead to a quick elimination of leukocytes (Griffith *et al*., 1995; Levy *et al*., 2015a) and participates in keeping the subretinal space physiologically devoid of immune cells (Gupta *et al*., 2003; Combadière *et al*., 2007; Lad *et al*., 2015). In diseases such as AMD, subretinal immunosuppression is impaired and macrophages (Mϕ) accumulate subretinally around the RPE. They are found on the apical side of the RPE adjacent to atrophic zones that define geographic atrophy (GA, a late form of AMD), around choroidal neovascularizations, and around large drusen, sub‐RPE debris deposits observed in early AMD (Gupta *et al*., 2003; Combadière *et al*., 2007; Lad *et al*., 2015; Levy *et al*., 2015a). Blood monocyte‐derived CD14^+^CCR2 + Mϕs invariably participate in this infiltration (Sennlaub *et al*., 2013).

We here used a coculture model of fresh human CD14^+^blood Mos and primary porcine RPE (pRPE) cells and a fully humanized system using human‐induced pluripotent stem cell (iPS)‐derived RPE (hiRPE) to mimic subretinal cellular interactions under physiological and pathological conditions. We demonstrate that TNF‐α, secreted from activated Mos, mediates downregulation of OTX2 and essential RPE genes such as *RDH5*. Our data show how subretinal, chronic inflammation can affect RPE function, which likely contributes to the visual dysfunctions and later degeneration in diseases such as AMD where subretinal Mϕs are observed.

## Results

### The RPE efficiently eliminates naïve, but not activated monocytes *in vitro*

**Figure 1:**
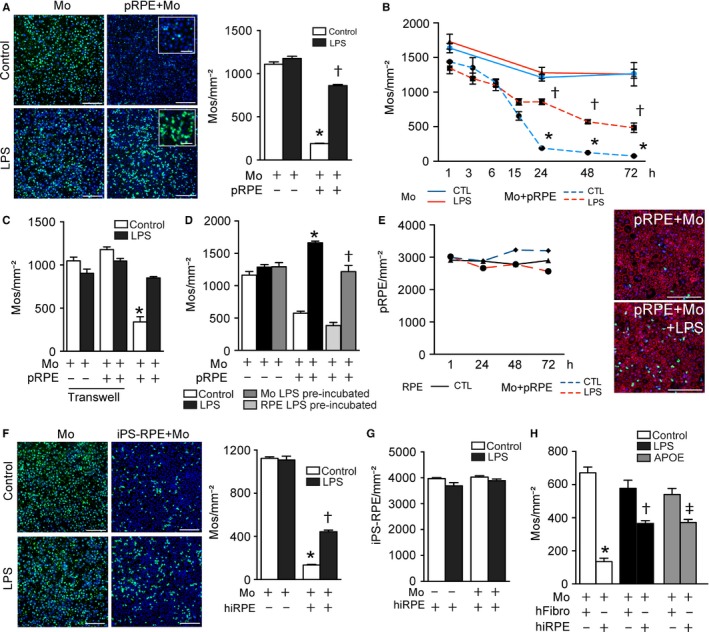
The RPE efficiently eliminates naïve‐ but not activated monocytes *in vitro*. (A) Representative pictures and quantification of CFSE labeled Mos at 24 h in monoculture (left panels) and pRPE/Mo coculture (right panels), incubated without (upper panels) or with LPS (lower panels). Blue fluorescence: DAPI; green fluorescence: CFSE (inset, close‐up view). Corresponding Arrayscan quantification of CFSE ^+^ DAPI ^+^Mos (n = 6/group; one‐way ANOVA/Bonferroni test: pRPE+Mos versus Mos *P ˂ 0.0001; pRPE+Mos+LPS versus pRPE+Mos †*P* ˂ 0.0001). (B) Quantification of the density of CFSE ^+^ DAPI ^+^Mos in monoculture (uninterrupted lines) or pRPE/Mo coculture (dotted lines) at indicated times with (red lines) or without (blue lines) LPS (n = 3/group; one‐way ANOVA/Bonferroni test: pRPE+Mos versus Mos *P < 0.0001 for each time point; pRPE+Mos+LPS versus pRPE+Mos at 24 h†*P* < 0.0001, at 48 h †*P* < 0.0001, at 72 h †*P* = 0.0064). (C) Quantification at 24 h of the density of CFSE ^+^ DAPI ^+^Mos in monoculture, and transwell or contact coculture with pRPE with and without LPS (n = 4/group; one‐way ANOVA/Bonferroni test: pRPE+Mos versus any other group *P ˂ 0.0001). (D) Quantification of the density of CFSE ^+^ DAPI ^+^Mos in monoculture or coculture after LPS‐pre‐activation of Mos or pre‐activation of the pRPE cells and 24 h culture in LPS‐free DMEM compared with LPS‐activated coculture (n = 3–4/group; one‐way ANOVA/Bonferroni test: pRPE+Mos+LPS versus pRPE+Mos *P ˂ 0.0001; pRPE+Mos/LPS‐PA versus pRPE+Mos†*P* = 0.0003). (E) Quantification of the density of OTX2^+^ DAPI ^+^ pRPE in monoculture (black uninterrupted line) or coculture (dotted lines) at 1 h, 24 h, 48 h, and 72 h with (red line) or without (blue line) 1 ng mL^−1^ of LPS (n = 4/group). Representative images of ZO‐1 (red), CFSE (green), DAPI (blue) staining of 24 h pRPE/Mo cocultures without (upper panel) and with LPS (lower panel). (F) Representative pictures and quantification of the density of CFSE ^+^ DAPI ^+^Mos at 24 h in monoculture (left panels) and hiRPE/Mo coculture (right panels), incubated without (upper panels) or with (lower panels) LPS. Blue fluorescence: DAPI; green fluorescence: CFSE (n = 6/per group; one‐way ANOVA/Bonferroni test: hiRPE+Mos versus Mos *P ˂ 0.0001; hiRPE+Mos+LPS versus hiRPE+Mos †*P *˂ 0.0001). (G) Quantification of the density of OTX2^+^ DAPI ^+^hiRPE at 24 h in monoculture or coculture with or without LPS (n = 6/group one‐way ANOVA/Bonferroni test: no significant). (H) Quantification of the density of CFSE ^+^ DAPI ^+^Mos after 24 h of coculture of Mo with either human foreskin fibroblasts, or hiRPE derived from these fibroblasts activated with LPS or APOE (n = 5/group; one‐way ANOVA/Bonferroni test: hiRPE+Mos versus hFibro+Mos *P ˂ 0.0001; hiRPE+Mos versus hiRPE+Mos+LPS; †*P* < 0.0001; hiRPE+Mos versus hiRPE+Mos+APOE ‡*P* < 0.0001).Data information: All experiments were repeated three times with similar results. Mos: monocytes; pRPE: porcine primary retinal pigment epithelial cells; hiRPE: human‐induced pluripotent stem cell‐derived RPE cells; hFibro: human foreskin fibroblasts; CTL: control; LPS: lipopolysaccharide; APOE: apolipoprotein E isoform 3. Scale bar A, E and F: 100 μm, insets 20 μm.

Physiologically, mononuclear phagocytes (MPs) are quickly eliminated when injected subretinally due to RPE FasL expression *in vivo* (Levy *et al*., 2015a). Mos have previously been shown to induce RPE apoptosis, in conditions where their number exceeds the number of RPE cells (Yang *et al*., 2009, 2011), which might correspond more to an autoimmune chorioretinitis type of setting. We here first cultured human CFSE‐stained, blood‐derived, magnetic bead‐sorted CD14^+^Mos alone (Fig. 1A left panels and B uninterrupted lines) or in coculture in a 0.5/1 ratio (as determined by cell counts after 1 h of coculture) with confluent porcine primary RPE cells (pRPE, Fig. 1A right panels and B dotted lines) for up to 3 days. The cultures were either conducted in Dulbecco's modified Eagle's serum (DMEM) alone (Fig. 1A upper panels and B blue lines) or containing lipopolysaccharide (LPS) (Fig. 1A lower panels and B red lines) to simulate a pro‐inflammatory micro‐environment. After 24 h, we observed a strong reduction in green‐fluorescent CFSE^+^Mos in the coculture condition that was significantly inhibited by LPS (Fig. 1A). LPS also induced a marked morphological change in the CFSE^+^Mos that appeared larger and stellate, compared with the control condition (Fig. 1A insets). At 24 h, automated counting of CFSE^+^DAPI^+^Mos revealed a significant 80% contraction of the Mo population in the coculture condition compared with the monoculture and the coculture at 1, 3, and 6 h (Fig. 1B). The reduction further decreased at 48, and 72 h (Fig. 1B). LPS did not significantly change the number of Mos in monoculture, or in the first 6 h of coculture, but the Mos resisted their elimination significantly when LPS was added to the coculture after 15 h, when a severe drop in naïve Mo numbers was observed. TUNEL staining of the coculture system did not reveal significant TUNEL^+^ nuclei numbers, likely because dying Mos detached from the RPE monolayer. However, transfer of any 24 h coculture supernatants to fresh wells did not lead to any cell adhesion, suggesting that the coculture did not contain any floating viable cells (data not shown).

We have previously shown that the RPE induces Mo apoptosis via FasL *in vivo* (Levy *et al*., 2015a)*,* which necessitates a cell–cell contact. We next cultured Mos in the upper chamber of a transwell with or without an RPE monolayer in the lower chamber or in the contact coculture as described above. Quantification of CFSE^+^DAPI^+^Mos at 24 h revealed no differences in cell density induced by the presence of RPE when Mos were cultured without contact in the transwells with or without LPS, contrary to contact cocultures (Fig. 1C), indicating that RPE/Mo contact is necessary for the Mo elimination. We next separately pre‐incubated the monocytes (1 h) and RPE cells (24 h) with LPS (40 ng mL^−1^) before the coculture in LPS‐free DMEM. Quantification of CFSE^+^DAPI^+^Mo density after 24 h of coculture shows that pre‐activation of Mos, but not pre‐activation of the RPE, significantly reduced RPE‐induced Mo elimination (Fig. 1D), similar to the condition when LPS was directly added to the coculture. The number of OTX2^+^DAPI^+^RPE nuclei, counted automatically by Arrayscan of OTX2 immunostained cultures, did not vary significantly between RPE monoculture and RPE/Mo coculture, be it with or without LPS (Fig. 1E). Counting of RPE cells using a zonula occludens 1 (ZO‐1) stain that delimitates the RPE cell borders gave similar results (not shown) and showed no major morphological RPE changes in these conditions (Fig. 1E picture panels).

The above‐described experiments were carried out with primary porcine RPE, which has the advantage to use primary RPE cells. To evaluate whether any of the results were due to interspecies incompatibilities in the coculture of human monocytes with porcine RPE cells, we next used human iPS‐derived RPE (hiRPE) cells in a purely human coculture for 24 h (Fig. 1F). Similar to the results obtained with primary porcine RPE, hiRPE induced a strong contraction of human CFSE^+^Mo numbers at 24 h compared with Mo monocultures and this reduction was significantly inhibited by LPS (Fig. 1F). Again the number of OTX2^+^DAPI^+^RPE nuclei evaluated by OTX2 immunostaining showed no difference of RPE nuclei in any condition (Fig. 1G). We next compared Mo survival in cocultures with either hiRPE cells or human primary fibroblasts (hFibro) from which the hiRPE cells were derived. Twenty‐four h of coculture clearly demonstrates that the transformation of hFibro into hiRPE cells induces the immunosuppressive phenotype (Fig. 1H). Accordingly, LPS induced an increase in Mo numbers in coculture with hiRPE but not with hFibro. To test whether a more AMD‐relevant Mo stimulation induces a similar resistance to elimination, we added apolipoprotein E (APOE) to the coculture system. We previously showed that APOE, which is observed in subretinal MPs of AMD patients (Levy *et al*., 2015a) and in MPs that express the AMD‐risk isoform APOE2 (Levy *et al*., 2015b), activates the CD14/TLR2/4‐dependent innate immunity receptor cluster and induces inflammatory cytokines (Levy *et al*., 2015a,b). Similar to LPS, recombinant APOE (but not heat‐inactivated APOE, data not shown) induced a robust increase in Mo numbers in coculture with hiRPE but not with hFibro (Fig. 1H).

Taken together, our coculture conditions closely mimic the RPE immunosuppressive effect on leukocytes observed *in vivo* (Griffith *et al*., 1995; Levy *et al*., 2015b). The activation of Mos significantly prolonged their survival in contact with porcine and human RPE cells similar to AMD where MPs are observed on the apical side of RPE cells around geographic atrophy lesions, subretinal neovascularization, and large drusen (Gupta *et al*., 2003; Combadière *et al*., 2007; Lad *et al*., 2015; Levy *et al*., 2015a).

### Activated Mos downregulate RPE *OTX2* expression

**Figure 2:**
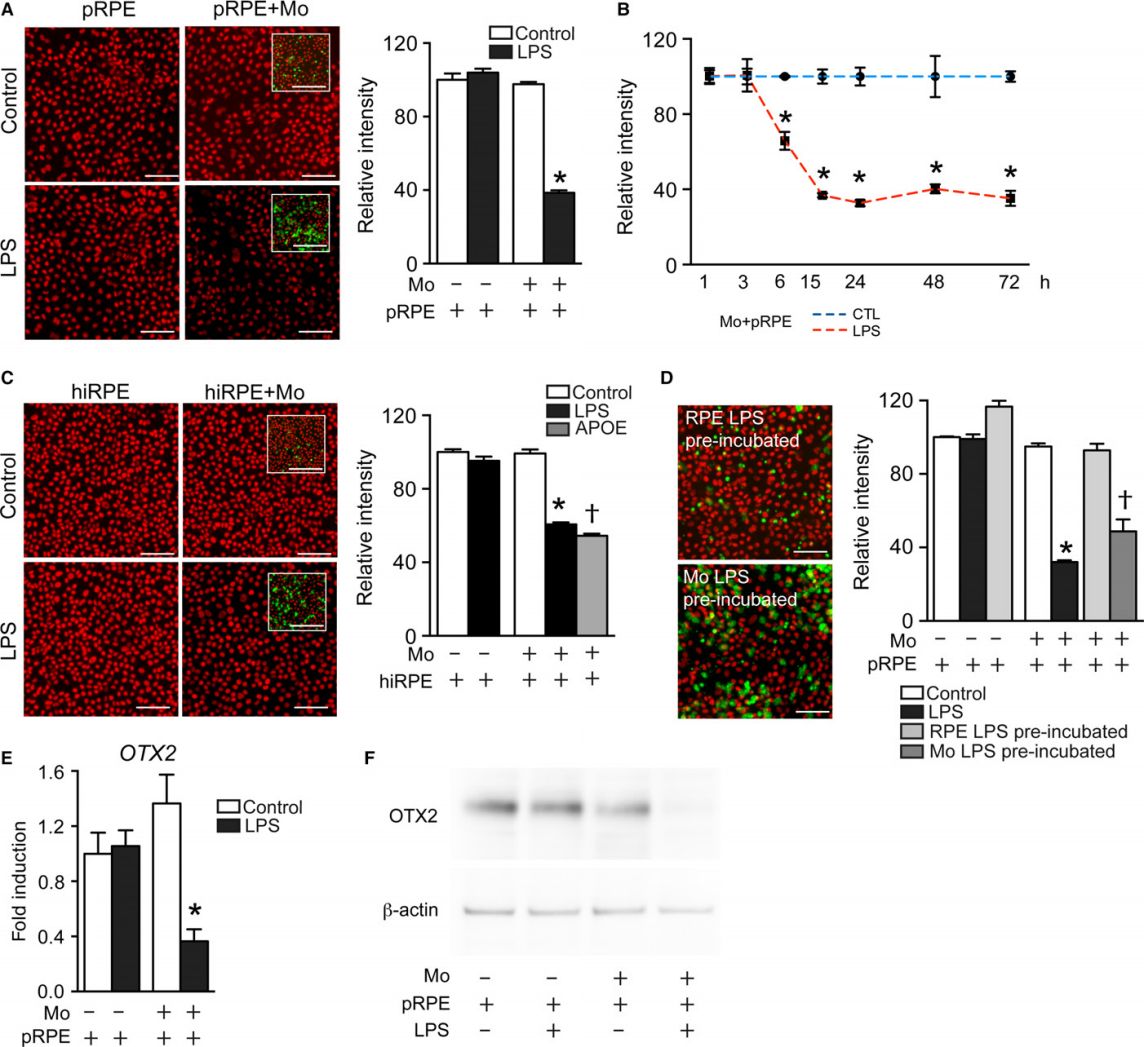
Activated Monocytes downregulate RPE OTX2 expression. (A) Representative pictures of OTX2 immunohistochemistry of pRPE at 24 h in pRPE cultures (left panels) and pRPE/Mo cocultures (right panels), incubated without (upper panels) or with (lower panels) LPS. Red fluorescence: OTX2; green fluorescence: CFSE (inset, close‐up view). Corresponding relative fluorescence intensity quantification of OTX2 fluorescence intensity (n = 6/group; one‐way ANOVA/Bonferroni test: pRPE+Mos+LPS versus any other group *P ˂ 0.0001). (B) Relative fluorescence intensity quantification at indicated times of OTX2 fluorescence intensity in cocultures with (red line) or without (blue line) LPS (n = 3/group; one‐way ANOVA/Bonferroni test: pRPE+Mos versus pRPE+Mos+LPS *P < 0.0001 for each time point). (C) Representative pictures of OTX2 immunohistochemistry of hiRPE at 24 h in monoculture (left panels) and hiRPE/Mo cocultures (right panels), incubated without (upper panels) or with (lower panels) of LPS. Red fluorescence: OTX2; green fluorescence: CFSE (inset, close‐up view). Corresponding relative fluorescence intensity quantification of OTX2 fluorescence intensity (n = 6/group, one‐way ANOVA/Bonferroni test: hiRPE+Mos+LPS versus any other group *P ˂ 0.0001). (D) Representative pictures of OTX2 immunohistochemistry (red) of cocultures of LPS‐pre‐activated pRPE (upper panel), or LPS‐pre‐activated Mos (lower panel) before 24 h of LPS‐free coculture in DMEM (green fluorescence: CFSE). Relative fluorescence intensity quantification at 24 h of OTX2 fluorescence intensity in control, LPS‐activated, and LPS‐pre‐activated pRPE and Mo cocultures (n = 3‐4/group, one‐way ANOVA/Bonferroni test: pRPE+Mos+LPS versus pRPE+Mos *P ˂ 0.0001; pRPE+LPS‐pre‐activated Mos versus pRPE+Mos †*P* ˂ 0.0001). (E) Relative expression of OTX2‐mRNA normalized with S26 expression quantified by RT‐qPCR of 24 h pRPE culture or pRPE+Mo coculture, with or without LPS (n = 8/group one‐way ANOVA/Bonferroni test: pRPE+Mos+LPS versus any other group *P ˂ 0.05). (F) Western blot analysis of OTX2‐ and β‐actin protein after 24 h of the indicated culture conditions. Data information: All experiments were repeated three times with similar results. Mos: monocytes; pRPE: porcine primary retinal pigment epithelial cells; hiRPE: human‐induced pluripotent stem cell‐derived RPE cells; CTL: control; LPS: lipopolysaccharide. Scale bar A, C, and D: 50 μm, insets 100 μm.

*OTX2* expression is maintained in the adult RPE where it regulates the expression of a number of essential RPE genes (Béby *et al*., 2010; Housset *et al*., 2013). Using OTX2 immunohistochemistry to positively identify RPE nuclei in the coculture experiments, we were able to accurately count the number of OTX2^+^DAPI^+^RPE nuclei, but also noticed that the intensity of the staining varied depending on the culture condition: The OTX2‐staining intensity (Fig. 2A, red staining) was similar in pRPE monocultures without or with LPS (Fig. 2A, left panels) and similar to the Mo/pRPE coculture condition without LPS after 24 h (Fig. 2A upper right panel). However, when LPS was added to the coculture condition, the intensity of OTX2 fluorescence was greatly diminished (Fig. 2A, lower right panel). Quantitative analysis of the fluorescence intensity using the Arrayscan showed that OTX2‐staining intensity was comparable in pRPE monoculture and Mo/pRPE cocultures at 24 h, except in the LPS coculture condition in which a very significant decrease was measured (Fig. 2A, graphic). This decrease was observed as soon as 6 h and throughout the experiment (Fig. 2B). Similarly, OTX2 fluorescence intensity was significantly diminished in human hiRPE cells after 24 h LPS‐ and APOE‐stimulated cocultures compared with unstimulated cocultures and hiRPE cell monocultures (Fig. 2C). When we pre‐incubated the monocytes and pRPE cells with LPS separately before their 24 h coculture, OTX2 immunofluorescent intensity again was significantly diminished by pre‐activation of Mos, but not the pRPE, similar to the condition when LPS was directly added to the coculture (Fig. 2D). OTX2 mRNA quantification by RT‐qPCR (Fig. 2E) and OTX2 protein by Western blot analysis (Fig. 2F) confirmed a significant reduction in OTX2 expression in the LPS coculture condition. The reduction in well above 50% of OTX2 expression observed in RT‐qPCR and Western blot analysis is unlikely due to the dilution by surviving Mos in the coculture, as they only represent maximally 25% (1000 Mos mm^−2^; 3000pRPE mm^−2^; Fig. 1) of the cells in the LPS coculture condition.

In summary, our data demonstrate that RPE OTX2 expression in the coculture condition is inversely proportional to Mo survival. The fact that LPS had no effect on OTX2 expression in RPE monocultures, but that LPS‐pre‐activated Mos did induce the downregulation shows that the activated Mos downregulate RPE OTX2 expression.

### TNF‐α mediates activated Mo‐induced RPE OTX2 downregulation

**Figure 3:**
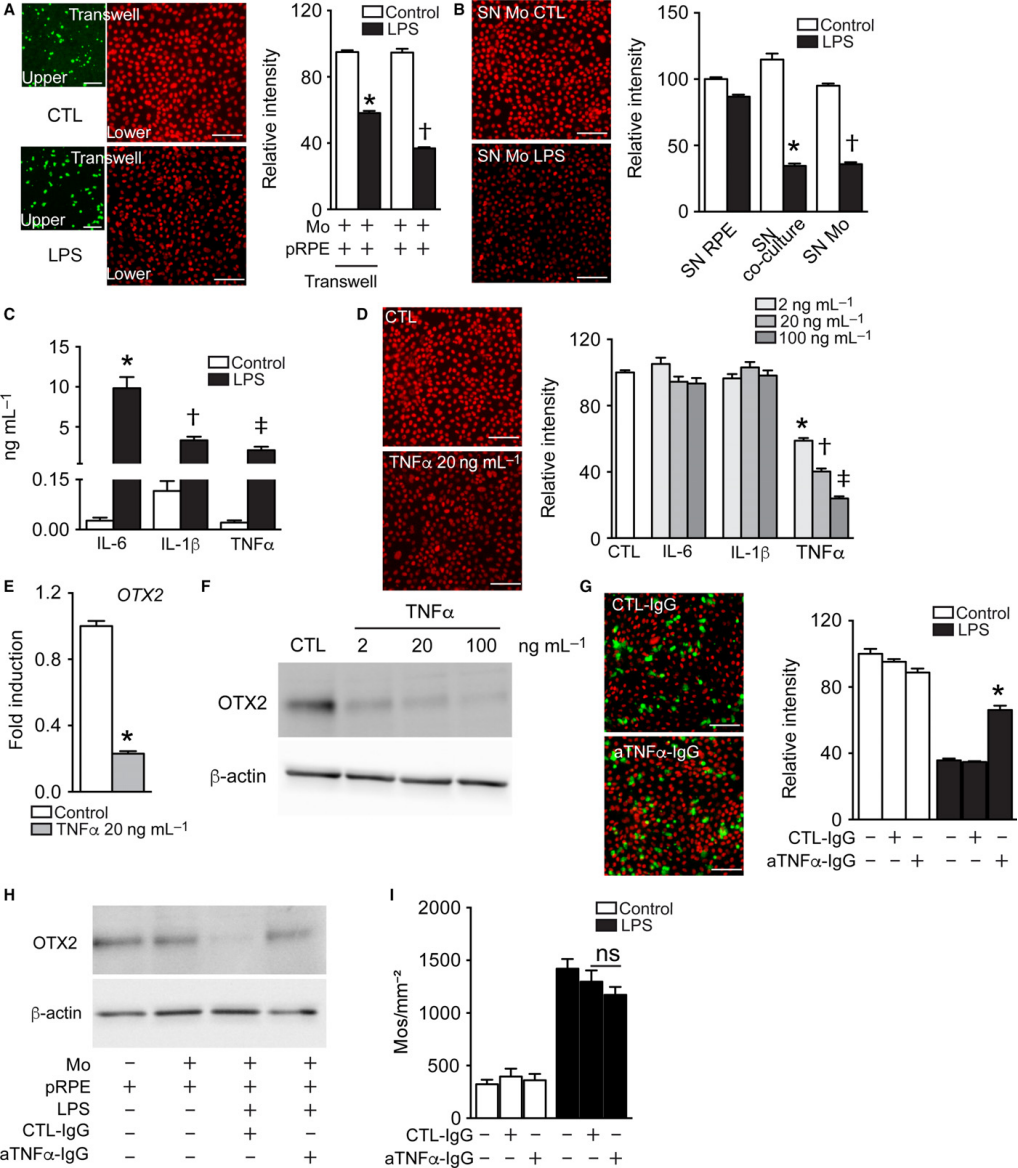
TNFα mediates activated Mo‐induced RPE OTX2 downregulation. (A) Representative images of the upper (green CFSE‐stained Mos) and lower chambers (OTX2 immunohistochemistry of pRPE, red) of 24‐h transwell cocultures and relative red fluorescence intensity quantification at 24 h (n = 4/group, one‐way ANOVA/Bonferroni test: TW pRPE+Mos+LPS versus any other TW group *P ˂ 0.0001; pRPE+Mos+LPS versus any other contact coculture group †*P* ˂ 0.0001). (B) Representative images (OTX2 red) and relative fluorescence intensity quantification of OTX2‐fluorescence intensity in pRPE culture after 24 h of incubation with conditioned supernatant (SN) from 24 h culture of pRPE, Mo/pRPE, or Mos with and without LPS. (n = 6/group, one‐way ANOVA/Bonferroni test: SN‐pRPE+Mos+LPS versus SN‐pRPE+Mos *P ˂ 0.0001; SN‐Mos+LPS versus SN‐Mos †*P* ˂ 0.0001). (C) IL‐6, IL‐1β and TNF‐α ELISA of supernatants from 24 h Mo culture with or without of LPS (n = 8–12/group, Mann–Whitney U‐test: IL‐6 *P = 0.0002; IL‐1β †*P* = 0.002; TNF‐α ‡*P* < 0.0001). (D) Representative pictures and relative fluorescence intensity quantification at 24 h of OTX2‐fluorescence intensity in pRPE culture incubated with IL‐6, IL‐1β, and TNF‐α at different concentration (2 ng mL^−1^, 20 ng mL^−1^, and 100 ng mL^−1^) compared with the control condition with no treatment (n = 6–9/group one‐way ANOVA/Bonferroni test: pRPE control versus any TNF‐α group *, †, ‡ *P* ˂ 0.0001). Red fluorescence: OTX2. (E) Relative expression of OTX2‐mRNA normalized with S26 expression quantified by RT‐qPCR of 24 h pRPE culture treated or not with 20 ng mL^−1^ of TNF‐α (n = 12/group Mann–Whitney U‐test: pRPE+ TNF‐α versus pRPE *P < 0.0001). (F) Western blot analysis of OTX2‐ and β‐actin protein after 24 h of pRPE culture treated with TNF‐α at 2, 20, and 100 ng mL^−1^. (G) Representative images and relative fluorescence intensity quantification at 24 h of OTX2‐immunohistochemistry (red) in pRPE/CFSE ^+^Mo (green) coculture incubated with and without LPS and anti‐TNF‐α‐ or control‐IgG (n = 4–6/group, one‐way ANOVA/Bonferroni test: pRPE+Mos+LPS+TNF‐α versus pRPE+Mos+LPS+IgG *P ˂ 0.0001). (H) Western blot analysis of OTX2‐ and β‐actin protein after 24 h of pRPE/Mo coculture incubated with anti‐TNF‐α‐ or control‐IgG. (I) Quantification of the density of CFSE ^+^ DAPI ^+^Mos at 24 h of pRPE/CFSE ^+^Mo coculture incubated with and without LPS and with anti‐TNF‐α‐ or control‐IgG (n = 4–6/group). Data information: All experiments were repeated twice with similar results. Mos: Monocytes; pRPE: porcine primary retinal pigment epithelial cells; CTL: control; LPS: lipopolysaccharide. Scale bars = 50 μm.

To determine whether a diffusible factor secreted from the activated Mos was responsible for the RPE OTX2 downregulation, we first quantified OTX2 immunofluorescence intensity in coculture conditions where the Mos were placed in the upper chamber of a transwell insert. As described for Fig. 1C, the noncontact coculture condition leads to stable monocyte counts with and without LPS. Interestingly, OTX2 fluorescence intensity was significantly diminished in LPS‐stimulated compared with nonstimulated transwell cultures and similar to the contact coculture condition (Fig. 3A), suggesting the existence of a diffusible factor. Indeed, supernatants from 24 h LPS‐stimulated Mo/RPE cocultures, or Mo monocultures diminish OTX2 fluorescence intensity in pRPE culture compared with supernatants from unstimulated cocultures and Mo monocultures (Fig. 3B). Taken together, these results strongly suggested that a Mo‐derived, LPS‐induced, secreted, diffusible factor is responsible for the OTX2 downregulation.

Upon LPS stimulation, Mos secrete a variety of inflammatory cytokines, such as IL‐1β, IL‐6, and TNF‐α that can have a profound effect of neurons and stromal cells (Hanisch, 2002). In our Mo culture conditions, the 24‐h LPS stimulation leads to IL‐1β, IL‐6, and TNF‐α concentrations of 2–10 ng mL^−1^ (Fig. 3C). As the secreted cytokines undergo partial degradation during 24 h, we next tested whether recombinant human IL‐1β, IL‐6, and TNF‐α at concentrations of 2, 20, and 100 ng mL^−1^ influenced OTX2 fluorescence intensity after 24 h of pRPE culture (Fig. 3D). Quantifications show that TNF‐α strongly and dose dependently decreased the fluorescence intensity of OTX2, while IL‐1β and IL‐6 induced no difference. Cotreatment with TNF‐α, IL‐6, and IL‐1β did not show any additional effect compared with TNF‐α monotreatment (data not shown). *OTX2* mRNA quantification by RT‐qPCR (Fig. 3E) and OTX2 protein by Western blot analysis (Fig. 3F) confirmed the TNF‐α strongly represses OTX2 expression. Next, we added an anti‐TNF‐α‐blocking antibody to the Mo/pRPE coculture, which significantly inhibited the LPS‐induced OTX2 fluorescence intensity downregulation compared with the control‐IgG (Fig 3G). Western blot analysis confirmed the partial restoration of OTX2 expression using the anti‐TNF‐α antibody (Fig. 3H). Interestingly, TNF‐α inhibition in LPS‐activated Mo/pRPE cocultures did not affect RPE immunosupressivity (Fig. 3I).

Taken together, our results show that TNF‐α, secreted from activated Mos, is sufficient to downregulate OTX2 in the RPE. Our experiments using an anti‐TNF‐α‐blocking antibody demonstrate that TNF‐α participates importantly in the RPE OTX2 downregulation observed in the activated Mo/RPE coculture.

### Activated Mos lead to OTX2‐dependent RPE gene downregulation

**Figure 4:**
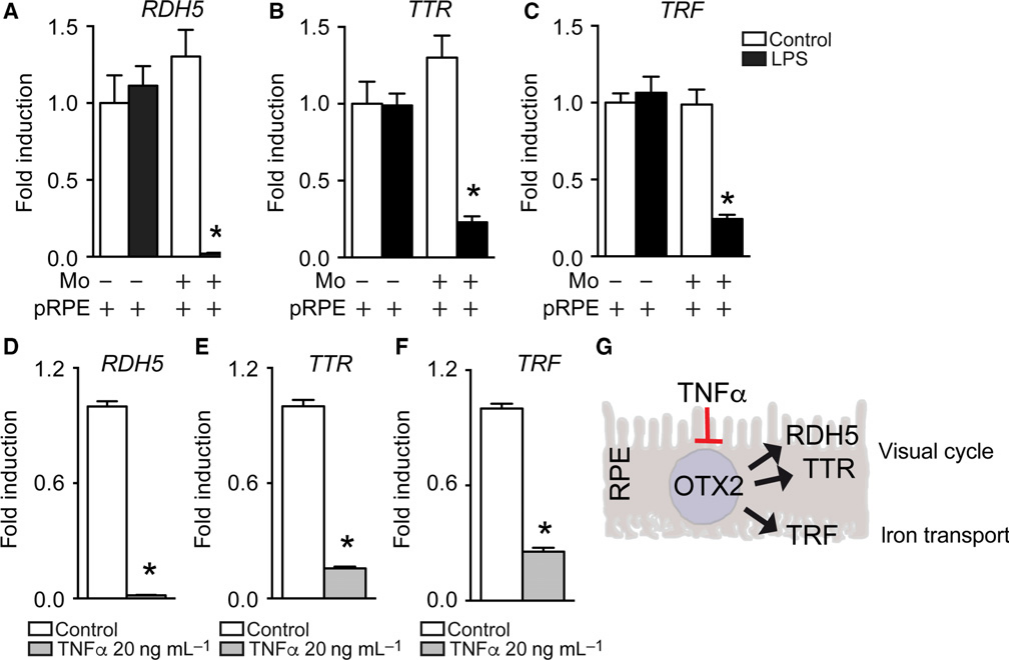
Activated Monocytes lead to essential OTX2‐dependent RPE gene downregulation. (A–C) Relative expression of (A) RDH5‐mRNA, (B) TTR‐mRNA, and (C) TRF‐mRNA normalized with S26 gene expression quantified by RT‐qPCR in 24‐h pRPE monoculture or pRPE+Mo coculture, with or without LPS (n = 6/group one‐way ANOVA/Bonferroni test: pRPE+Mos+LPS versus any other group *P ˂ 0.0001). (D–F) Relative expression of (D) RDH5‐mRNA, (E) TTR‐mRNA, and (F) TRF‐mRNA normalized with S26 gene expression quantified by RT‐qPCR in 24 h pRPE culture with or without 20 ng mL^−1^ of TNF‐α (n = 6/group Mann–Whitney U‐test: pRPE+TNF‐α versus pRPE *P = 0.0022). (G) Graphical summary. Data information: All experiments were repeated twice with similar results. Mos: Monocytes; pRPE: porcine primary retinal pigment epithelial cells; CTL: control; LPS: lipopolysaccharide.

OTX2 regulates RPE genes that are crucially involved in homeostasis and notably the RPE part of the visual cycle (Housset *et al*., 2013; Masuda *et al*., 2014). We next analyzed the expression of TTR and RDH5, both essential for the visual cycle (Cavallaro *et al*., 1990; Yamamoto *et al*., 1999; Cideciyan *et al*., 2000), and TRF, an iron transport protein (He *et al*., 2007; Picard *et al*., 2015). mRNA quantification by RT‐qPCR shows that TTR, TRF, and RDH5 transcription levels are not affected by LPS or the presence of Mos alone, but all three tested genes are severely downregulated in the LPS‐activated Mo/RPE coculture (Fig. 4A‐C). Again, incubation with recombinant TNF‐α was sufficient to severely reduce the transcription levels of TTR, TRF, and RDH5 quantified by RT‐qPCR (Fig. 4D–F).

In summary, these results show that the reduced OTX2 expression induced by activated Mos or recombinant TNF‐α is associated with a grave downregulation of the essential RPE genes it regulates (Fig 4G).

## Discussion

*In vivo*, the retinal pigment epithelium (RPE) is a particularly immunosuppressive tissue as it quickly eliminates adjacent leukocytes through multiple mechanisms that include FasL/FAS signaling (Griffith *et al*., 1995; Levy *et al*., 2015a). In fact, RPE allografts to nonimmune privileged sites (kidney capsule) of nonimmunocompromised, nonhistocompatible recipients, efficiently kill approaching immune effector cells (T cells, macrophages) and survive prolonged periods of time despite the induction of an adoptive immune response to the grafted cells (Wenkel & Streilein, 2000). Physiologically the RPE immunosuppressive capacities likely contribute to the anatomical particularity that the subretinal space, delimitated by the RPE and the photoreceptor outer segments, is not only devoid of leukocytes in general, but also of resident microglial cells that are only located in the inner retina (Gupta *et al*., 2003; Combadière *et al*., 2007; Lad *et al*., 2015). Our coculture of blood‐derived CD14^+^Mos and RPE closely mimics this immunosuppressive effect *in vitro*, as the number of Mos decreases dramatically between six and 24 h after they enter in contact with the RPE, similar to subretinally injected Mos *in vivo* (Levy *et al*., 2015a). We show that a cell–cell contact is necessary for their elimination, as the Mos were not eliminated if cultured in a noncontact scenario using transwell plates. Activation of the coculture by LPS very significantly inhibited the RPE‐induced Mo elimination. The fact that pre‐activation of Mos by LPS, but not the pre‐activation of the RPE, led to a similar inhibition of Mo elimination clearly showed that the effect was due to the Mo activation. Interestingly, APOE, which we showed is strongly expressed in subretinal MPs in AMD, induces the secretion of inflammatory cytokines via the activation of the innate immunity receptor cluster and prolongs the survival of subretinally injected MPs *in vivo* (Levy *et al*., 2015a), had a similar effect than LPS. The resistance to elimination could either be due to an intrinsic resistance to RPE‐induced cell death of activated Mo or the activated Mos might alter the immunosuppressive capacities of the RPE.

Human Mo cocultures with primary porcine RPE and human iPS‐derived RPE cells revealed similar results throughout the study, showing that possible interspecies incompatibilities had no effect on the observed phenotype.

Contrary to previous studies in which primary human RPE cells were used (Elner *et al*., 2003; Yang *et al*., 2009, 2011), CD14^+^Mos never induced pRPE or hiRPE apoptosis or major morphological changes in our coculture conditions. Even higher Mo/RPE cell ratios of LPS‐activated CD14^+^Mos failed to induce RPE apoptosis in our hands (data not shown). These differences might be due a more pronounced susceptibility of primary human RPE from aged donors (50–86 years) (Yang *et al*., 2011) compared with primary porcine RPE (prepared from young pigs) and hiRPE. However, we did observe that increased survival of Mos in contact with the RPE, either in activated cocultures or in RPE cultures with pre‐activated Mos, invariably led to a strong decrease in RPE‐OTX2 expression. We observed this diminution by quantitative OTX2 immunofluorescence intensity measurements, in RT‐qPCRs and in Western blot analysis. Our data show that LPS had no direct effect on *OTX2* expression in RPE, but that LPS‐pre‐activated Mos did induce the downregulation whether in contact or in noncontact transwell cultures. We show that TNF‐α, secreted from activated Mos, is sufficient to downregulate OTX2 in the RPE and that an anti‐TNF‐α‐blocking antibody partially restores its expression in the activated Mo/RPE coculture. OTX2 expression is regulated by β‐catenin during development (Westenskow *et al*., 2009). TNF‐α has previously been shown to induce Dkk1, which inhibits WNT‐dependent β‐catenin nuclear translocation (Diarra *et al*., 2007; Nava *et al*., 2010). TNF‐α might thereby inhibit β‐catenin‐dependent OTX2 downregulation in the RPE.

OTX2 is a key transcription factor in the adult RPE where it regulates the expression of a number of essential genes involved in RPE melanogenesis, iron transport, and the retinol visual cycle. We here show that the observed alterations in OTX2 expression, under LPS‐induced inflammatory conditions or by recombinant TNF‐α, are associated with a grave downregulation of essential RPE genes it regulates, such as *TRF*,* TTR*, and *RDH5*. TTR and RDH5, together with RPE65 and LRAT, are necessary to re‐isomerize all‐trans‐retinal into 11‐cis‐retinal essential for rhodopsin function. Dysfunction of the RPE part of the visual cycle leads to an increase in recovery time in the scotopic ERG after bleach, as it takes more time to reproduce 11‐cis‐retinal, exemplified in RDH5‐deficient patients (Cideciyan *et al*., 2000). Interestingly, early AMD patients also present a marked increase in recovery time after bleach (Owsley *et al*., 2001; Flamendorf *et al*., 2015). These functional differences appear before significant loss of RPE and likely reflect a diminished efficiency of the RPE component of the visual cycle. In our study, we used LPS, which is unlikely to play a direct role in AMD, but also APOE, which we showed is strongly expressed in subretinal MPs in AMD (Levy *et al*., 2015a), to activate the Mos *in vitro*. TNF‐α can be induced by diverse stimuli and patients with the CFH AMD‐risk variant are associated with higher systemic levels of TNF‐α (Cao *et al*., 2013), and patients with monocytes that express the greatest amount of TNF‐α have higher prevalence of choroidal neovascularization (Cousins, 2004). Others and we have previously shown that large drusen (that characterize early AMD) are foci of subretinal MP accumulation (Sennlaub *et al*., 2013; Lad *et al*., 2015; Levy *et al*., 2015a). Even though there is not yet direct evidence for OTX2 downregulation, it is tempting to speculate that TNF‐α, produced by infiltrating MPs, induces a slowing of the visual cycle in early AMD, before RPE lesions become evident.

Interestingly, inflammation/TNF‐α‐dependent OTX2 downregulation might also have implications in congenital microphthalmia and auditory defects, common after intrauterine infections, notably with cytomegalovirus (CMV) (Becroft, 1981). CMV has been shown to potently induce TNF‐α (Smith *et al*., 1992), which in turn might downregulate OTX2 necessary for eye and ear development (Acampora *et al*., 1995; Cantos *et al*., 2000; Fossat *et al*., 2006).

Taken together, our study reveals a novel link between inflammation and OTX2‐related functions. In development, inflammation‐induced downregulation of OTX2 might participate in malformation, and in the adult it could explain RPE dysfunction in diseases such as early AMD. Therapeutic inhibition of inflammation or restoration of *OTX2* expression might help restore RPE function in retinal diseases with a subretinal inflammatory component.

## Experimental procedures

### Porcine RPE cell culture

Primary porcine RPE cells were isolated as previously described (Arnault *et al*., 2013). Porcine eyes were bought at a local slaughterhouse (Guy Harang, Houdan, France) in agreement with the local regulatory department and the slaughterhouse veterinarians. This procedure adheres to the European initiative for restricting animal experimentation because not a single animal was killed for our experimentation. Porcine eyes were obtained 2 or 3 h after enucleation in CO2‐independent serum (Thermo Fisher Scientific). Eyes were cleaned for muscles and immersed few minutes in an antiseptic solution (Pursept‐A Xpress, Merz Hygiene GmbH). Anterior segment of the bulb was removed, as well as lens, vitreous, and retina. Each eye cup was washed 2 times with PBS (Thermo Fisher Scientific) and incubated for 1 h at 37 °C with 0.25% trypsin–EDTA (Thermo Fisher Scientific). RPE cells were pipetted off the choroid and resuspended in Dulbecco's modified Eagle's serum (DMEM, Thermo Fisher Scientific) supplemented with 20% fetal calf serum (FCS, Thermo Fisher Scientific) and 1% antibiotics penicillin/streptomycin (PS). Purified cells were then seeded on a 60‐mm Petri dish in DMEM‐FCS20%‐PS1% and incubated with a controlled atmosphere at 5% CO2 at 37 °C. The culture medium was changed 24 h after the seeding. When cells were confluent, 0.05% trypsin–EDTA was added for 5 min at 37 °C to detach the RPE cells. They were finally seeded on 96‐well culture plate (Corning) at the concentration of 75 000 cells/well in DMEM‐FCS20%‐PS1%. Confluence state was obtained after 3 days in culture at 37 °C, 5% of CO_2_. Cells were used the fourth day.

### Human foreskin fibroblast and induced pluripotent stem cell‐derived RPE cells

Human iPS cells were derived from fibroblasts as previously described (Reichman *et al*., 2014) and cultivated on feeder cells that were cultivated without FGF‐2 for 48 h. Then, the ReproStem medium (Reprocell, Ozyme) was removed and replaced by a proneural medium (DMEM‐F12 supplemented with 1% N2 and 0.1% penicillin/streptomycin, Thermo Fisher Scientific). The medium was changed every 2 days until apparition of pigmented cells. Patches of pigmented cells were cut with a needle and coated in a Geltrex matrix (Thermo Fisher Scientific). Once confluence obtained, hiRPE cells were characterized by RT‐qPCR and immunocytochemistry using specific markers of RPE cells (Reichman *et al*., 2014). Cells were used after a maximum of two passages. In our study, human foreskin fibroblasts and RPE cells differentiated from three different human iPS‐RPE cells lines were used (Reichman *et al*., 2014).

### Human blood monocyte isolation

Human blood Mos from healthy donor were purified after written and informed consent in the Centre National d'Ophtalmologie des Quinze‐Vingts (Paris). Briefly, peripheral mononuclear cells from blood of healthy donor were obtained by Ficoll gradient centrifugation, washed three times, and were sorted with EasySep Human Monocyte Enrichment Cocktail without CD16 Depletion kit (StemCell Technologies). Purified monocytes were then labeled with 488‐nm Cell Trace CFSE (Thermo Fisher Scientific) and cultured.

### Coculture and treatments

The day before experiment, RPE cells were serum‐starved. A total of 100 000 freshly purified human monocytes were added to confluent fibroblasts or RPE cells in DMEM‐PS1%. Treatments were applied directly to the culture medium. To stimulate Mos, we used lipopolysaccharide (LPS) of *E.Coli* (Sigma‐Aldrich, 40 ng mL^−1^) or apolipoprotein E (APOE isoform 3, Leinco Technologies, 10 μg ML^−1^). In specific experiments, cells were incubated with the indicated recombinant human proteins (TNF‐α, IL‐6, and IL‐1β), obtained from R&D. Blocking goat anti‐human TNF‐α (R&D) and mouse anti‐human IgG (R&D) antibodies were used at 25 μg mL^−1^. Cells were incubated at 37 °C for 24 h or more for further experimentation. At the end of coculture, cells were washed two times with PBS and fixed in 4% paraformaldehyde (PAF) for 10 min. Alternatively, supernatants were removed and RA1 or RIPA lysis buffer was added on cells to perform real‐time polymerase chain reaction (RT‐PCR) or Western blot assays, respectively.

### Immunofluorescence microscopy

Fixed cells were washed twice in PBS and incubated for 2 min in a permeabilization solution (freshly prepared 0.1% triton and 0.1% sodium citrate in PBS). Cells were blocked for 1 h in PBS and triton 0.1% containing 5% horse serum (Thermo Fisher Scientific) and incubated overnight at 4 °C with the primary antibodies (polyclonal goat anti‐human OTX2, 1/500, R&D; monoclonal rat anti‐mouse ZO‐1, 1/300, Millipore) diluted in PBS triton 0.1% and 1% horse serum. Cultures were then incubated for 1 h at room temperature with the secondary antibody produced in Donkey (AlexaFluor 647 nm, 1/500, Thermo Fisher Scientific), and nuclei were counterstained with Hoechst (1/1000, Sigma‐Aldrich). Cells were washed twice in PBS and observed under fluorescent microscope (Arrayscan VTI HCS Reader, Thermo Fisher Scientific). Twenty‐five fields per well were analyzed and recorded by Arrayscan software (HCS iDev Cell Analysis Software, Thermo Fisher Scientific).

### Enzyme‐linked immunosorbent assay (ELISA)

To determine the amount of human TNF‐α, IL‐6, and IL‐1β in the supernatant of the cocultures, we used the human ELISA DuoSet kit (R&D Systems). Briefly, 96‐well plates were coated with a capture antibody overnight at room temperature (RT). After washing the wells, the plate was blocked in buffer containing bovine serum albumin (BSA) for 1 h. After a step of washes, samples or standards were added and incubated for 2 h. After washing, detection antibodies were added and incubated for an additional 2 h. Wells were then washed and a streptavidin–HRP solution was added for 20 min. After a final step of washes, substrate solution (mixture of H_2_0_2_ and tetramethylbenzidine) was added for 20 min. After addition of 50 μl of stop solution (H_2_SO_4_), the optical density of each well is measured at 450 nm using a spectrofluorometer (Tecan).

### RT‐PCR

Total RNA was isolated with Nucleospin RNAII (Macherey Nagel). RNA yields were then measured at 260 nm using the NanoDrop^™^ 8000 spectrophotometer. Typically, concentrations of RNA from our cell culture are comprised between 30 and 40 ng μL^−1^. Single‐strand cDNA was synthesized using 1 μg of total mRNA, pretreated with DNase amplification grade, using oligo‐dT as primer and superscript II reverse transcriptase (Thermo Fisher Scientific). For real‐time PCR, 1/100 of cDNA was incubated with the polymerase and the appropriate amounts of nucleotides (PowerSYBR Green PCR mix, Applied Biosystems). qPCR was realized by the StepOne Plus real‐time PCR system (Applied Biosystems). Results were normalized with expression of RPS26. PCRs were performed in 45 cycles of 15s at 95 °C, 45s at 60 °C. Primers for RT‐PCR were purchased from IDT. Sequences are as follows: OTX2 forward 5'‐CCCACTGTCAGATCCCTTGT‐3'; OTX2 reverse 5'‐GGGGACTGATTGAGATGGCT‐3'; RDH5 forward 5'‐GCTGAATGTGAACACGCTGG‐3'; RDH5 reverse 5'‐GAAGAAACCAGGCTCCACGA‐3'; TRF forward 5'‐TCCGCAGAAAACACCGAAGA‐3'; TRF reverse 5'‐ACACAGTTTTCACCCTCAGTT‐3'; TTR forward 5'‐TGGAAGGCACTTGGCATTTC‐3'; TTR reverse 5'‐GGTGGAGTAAGAGTAGGGGC‐3'; RPS26 forward 5'‐TCGATGCCTATGTGCTTCCC‐3'; RPS26 reverse 5'‐CAGCACCCGCAGGTCTAAAT‐3'.

### Western blotting

A dosage of the protein lysate was performed using the BiCinchoninic acid Assay (BCA) test with a standard range of BSA. Twenty micrograms of total cell lysates in SDS sample buffer with reducing agent was denaturated at 70 °C for 10 min and loaded on a 4–12% bis‐tris gel. Migration was done at 100V in running buffer, and proteins were transferred to a nitrocellulose membrane at 30V for 1 h30 in transfer buffer. Control of the transfer quality was done by coloration of the membranes with Red Ponceau dye before the saturation step with TBS‐T (25 mM Tris, 150 mM NaCl, Tween 20X 0.1%) containing 5% of skimmed milk. Membranes were washed 3 times with TBS‐T before incubation overnight with the primary antibody anti‐OTX2 (Polyclonal Goat anti‐human OTX2, 1/500, R&D), or anti‐β‐actin (monoclonal mouse anti‐β‐actin, 1/1000, Sigma‐Aldrich) at 4 °C. After three TBS‐T washes, membranes were incubated at room temperature for 1 h with the secondary antibody coupled to peroxidase (HRP). The revelation by chemiluminescence is done using the ECL prime kit reagent and pictures are obtained with a CCD camera (Fusion).

### Statistical analysis

Graph prism 6 (GraphPad Software) was used for data analysis and graphic representation. All values are reported as mean +/− standard error of the means (SEM). Statistical analysis was performed by Mann–Whitney U‐test to compare 2 groups. When more groups had to be compared, one‐way ANOVA followed by Bonferroni post‐test was used (multiple comparison). *P* < 0.05 was considered statistically significant.

## Funding

This work was supported by grants from INSERM, ANR MACLEAR (ANR‐15‐CE14‐0015‐01), Labex Lifesenses, Carnot, and by the Association de Prévoyance Santé de ALLIANZ. This work was performed in the frame of the LABEX LIFESENSES [ANR‐10‐LABX‐65] supported by the ANR within the Investissements d'Avenir program [ANR‐11‐IDEX‐0004‐02].

## Conflict of interest

None declared
